# A Proteomic Study for the Discovery of Beef Tenderness Biomarkers and Prediction of Warner–Bratzler Shear Force Measured on *Longissimus thoracis* Muscles of Young Limousin-Sired Bulls

**DOI:** 10.3390/foods10050952

**Published:** 2021-04-27

**Authors:** Yao Zhu, Mohammed Gagaoua, Anne Maria Mullen, Alan L. Kelly, Torres Sweeney, Jamie Cafferky, Didier Viala, Ruth M. Hamill

**Affiliations:** 1 Food Quality and Sensory Science Department, Teagasc Food Research Centre, Ashtown, D15 KN3K Dublin 15, Ireland; yao.zhu@teagasc.ie (Y.Z.); gmber2001@yahoo.fr (M.G.); AnneMaria.mullen@teagasc.ie (A.M.M.); Jamie.Cafferky@teagasc.ie (J.C.); 2 School of Food and Nutritional Sciences, University College Cork, T12 K8AF Cork, Ireland; a.kelly@ucc.ie; 3 School of Veterinary Sciences, University College Dublin, D04 V1W8 Dublin 4, Ireland; torres.sweeney@ucd.ie; 4 Metabolomic and Proteomic Exploration Facility (PFEM), INRAE, F-63122 Saint-Genès-Champanelle, France; didier.viala@inrae.fr

**Keywords:** foodomics, beef tenderness, bovine biomarkers, muscle, proteome, liquid chromatography-tandem mass spectrometry (LC-MS/MS)

## Abstract

Beef tenderness is of central importance in determining consumers’ overall liking. To better understand the underlying mechanisms of tenderness and be able to predict it, this study aimed to apply a proteomics approach on the *Longissimus thoracis* (LT) muscle of young Limousin-sired bulls to identify candidate protein biomarkers. A total of 34 proteins showed differential abundance between the tender and tough groups. These proteins belong to biological pathways related to muscle structure, energy metabolism, heat shock proteins, response to oxidative stress, and apoptosis. Twenty-three putative protein biomarkers or their isoforms had previously been identified as beef tenderness biomarkers, while eleven were novel. Using regression analysis to predict shear force values, MYOZ3 (Myozenin 3), BIN1 (Bridging Integrator-1), and OGN (Mimecan) were the major proteins retained in the regression model, together explaining 79% of the variability. The results of this study confirmed the existing knowledge but also offered new insights enriching the previous biomarkers of tenderness proposed for *Longissimus* muscle.

## 1. Introduction

Meat-eating quality consists of a complex set of sensory traits including tenderness, flavour, and juiciness, each of which plays an important role in defining the appeal of beef to consumers [1,2]. Amongst these quality attributes, however, tenderness is considered to be one of the most important factors in purchase decisions regarding beef, with negative experience on toughness contributing to a lower likelihood of repeat purchase [3]. To meet the expectations of consumers, beef producers must pursue the provision of consistent high-quality beef. The underlying mechanisms involved in dictating the final meat tenderness are intricate, with muscle biochemistry interacting with processing, influenced by several factors including breed [4,5], gender [6], age at slaughter [7], muscle type [8,9], cooking temperature [5], stress at slaughter [10], and post-slaughter management and many other factors from farm-to-fork [9,11].

There have been a number of studies using omics tools to, firstly, enhance our understanding of the pathways and processes contributing to beef tenderness variation [12,13] and secondly, to propose prediction equations to explain the observed variability in this important quality trait [14]. Thus, omics-related analytical technologies and bioinformatics tools have been applied in recent decades, resulting in a deeper understanding of gene expression, physiological responses, and other metabolic processes that are involved in meat quality determination, especially tenderness [2,12,15].

Foodomics is an emerging group of disciplines encompassing genomics, transcriptomics, proteomics, metabolomics, and lipidomics applied to food and parameters related to its quality and has been extensively used to study both fresh meat and meat products [16]. Among the many foodomics approaches, proteomics played an important role in the discovery of candidate biomarkers of several meat quality attributes [2,13,17]. A pipeline to search for proteomic biomarkers of beef tenderness was proposed [12,14]. Compared with traditional evaluation methods for beef tenderness using instrumental or sensory methods, an optimised protocol for quality monitoring using rapid methods to record the abundance of specific proteins of interest would offer an advantage to predict the meat quality before consumption. Moreover, these approaches have the potential to be developed further to allow advanced prediction of the future tenderness phenotype at a range of stages from farm-to-fork [11,15].

This study aimed to apply shotgun proteomics on muscle tissue of young Limousin-sired bulls to identify putative biomarkers of beef tenderness evaluated by Warner–Bratzler shear force (WBSF) [15]. We further aimed to propose regression models and identify the main biological interactions among the proteins underpinning WBSF variation to gain insights into the mechanisms of beef tenderness determination.

## 2. Materials and Methods

### 2.1. Meat Sample Collection

Eighteen young Limousin-sired bulls were obtained and finished at the Irish Cattle Breeders Federation Progeny Test Centre and slaughtered in an EU-licensed abattoir by electrical stunning (50 Hz) followed by exsanguination from the jugular vein. All 18 animals were finished to U- to E+ conformation score, 3- to 5= fat score and at an average age of 487 days (±24 days) and live weight of 678 kg (±58 kg) [6]. According to the muscle sampling method used by Zhu et al. [18], *Longissimus thoracis et lumborum* (LTL) samples from the 10th rib of each carcass were collected and finely macerated in 5 mL RNAlater^®^ for 24 h. The RNAlater^®^ was then removed, and the sample was subsequently transferred for storage at −80 °C until analysis. Loins were boned out at 48 h post-mortem, and steaks with a thickness of 2.54 cm were cut out from the right-side LTL of the carcass starting at the anterior end and packaged in vacuum bags. The steaks were then aged for 14 days and stored at −20 °C until Warner–Bratzler shear force (WBSF) analysis.

### 2.2. Warner–Bratzler Shear Force Measurement

Steaks were thawed at room temperature by immersion in a circulating water bath for 4 h. After that, external fat was trimmed from the steaks, and they were cooked in open bags in a circulating water bath (Grant Instruments Ltd., Cambridge, UK) set at 72 °C to reach an internal end-point cooking temperature of 71 °C. The cooked steaks were cooled down and stored in a refrigerator at 4 °C overnight. Shear force analysis was conducted following a modified version of the guideline of the American Meat Science Association (AMSA) [6]. For each steak, seven cores were taken with a 1.27 cm diameter parallel to the muscle fibre direction. The shear force was measured by an Instron 4464 Universal testing machine (Instron Ltd., Buckinghamshire, UK), and data analysed using Bluehill 2 Software (Instron Ltd., Buckinghamshire, UK). To reduce the standard deviation among the cores, the maximum and minimum shear values (Newton) were discarded, and the mean values of the remaining 5 cores were reported.

### 2.3. Muscle Protein Extraction

Frozen muscle tissue samples (80 mg) were first homogenised in 2 mL of 8.3 M urea, 2 M thiourea, 1% dithiothreitol, 2% 3-[(3-cholamidopropyl) dimethylammonio]-1-propanesulfonate, 2% immobilised pH gradient (IPG) buffer pH 3–10 using a T 25 digital ULTRA-TURRAX^®^ following the protocol of Bouley et al. [19]. To remove non-extracted cellular components, fat, insoluble proteins, the protein homogenates were incubated with shaking for 30 min at 4 °C followed by a 30 min centrifugation at 10,000 × *g*. The supernatant was then transferred into Eppendorf tubes for protein quantification using the dye-binding protocol of Bradford [20].

### 2.4. Shotgun Proteomics

#### 2.4.1. One Dimensional SDS-PAGE and Protein Bands Preparation

The protein extract was firstly mixed (1:1) with Laemmli sample buffer (Bio-Rad Laboratories, Deeside, UK), then concentrated on 1D stacking gel of sodium dodecyl sulphate-polyacrylamide gel electrophoresis (SDS-PAGE) using commercial Mini-PROTEAN^®^ TGX™ precast gels of 8.6 × 6.7 × 0.1 cm and 12% polyacrylamide (Bio-Rad Laboratories, Deeside, UK). Twenty µg proteins were loaded in each gel lane, and the electrophoresis was run at 4 watts for about 15 min to concentrate the proteins in the stacking gel [21]. Subsequently, the gels were washed three times with Milli-Q water, stained with EZ Blue Gel staining reagent (Sigma, Saint Louis, MO, USA) with gentle shaking for 2 h, and then washed with Milli-Q water. The protein bands were excised from the washed gels using a sterile scalpel and immediately transferred into Eppendorf tubes containing 200 µL of 25 mM ammonium bicarbonate (Sigma, Saint Louis, MO, USA)-5% acetonitrile for 30 min. Then, bands were washed twice using 200 µL of 25 mM ammonium bicarbonate-50% acetonitrile for 30 min each. Finally, they were dehydrated with 100% acetonitrile for 10 min, and the liquid was discarded. Subsequently, the dried protein bands were stored at −80 °C until LC-MS/MS analysis. The immobilised proteins in the 1D gel bands were discoloured/reduced-alkylated, as described by Gagaoua et al. [22].

#### 2.4.2. LC-MS/MS

The hydrolysis of the protein bands was carried out with 48 µL of a 25 mM ammonium bicarbonate buffer-12.5 ng/µL trypsin solution (Promega) per band for 5 h in an oven at 37 °C. Then, 30 μL buffer was added periodically during hydrolysis so that the bands were always covered with liquid. The extraction of the peptides was carried out under ultrasound (15 min) with acetonitrile and trifluoroacetic acid. Then, the supernatant was transferred into 500 μL Eppendorf tubes and dry concentrated using a Speedvac for 2 h. The volume was adjusted exactly to 20 µL with a solution of isotopologic peptides (50 pmol/µL) that was diluted 18 times in a 0.05% Trifluoroacetic acid (TFA) solution. After passing through the ultrasonic bath (10 min), the entire supernatant was transferred to the High performance liquid chromatography (HPLC) vial before LC-MS/MS analysis.

For the separation, the hydrolysate was injected into the nano-LC-MS/MS (Thermo Fisher Scientific) using an Ultimate 3000 system coupled to a QExactive HF-X mass spectrometer (MS) with a nanoelectrospray ion source. Briefly, 1 μL of hydrolysate was first preconcentrated and desalted at a flow rate of 30 µL/min on a C18 pre-column 5 cm length × 100 µm (Acclaim PepMap 100 C18, 5 µm, 100 Å nanoViper) equilibrated with trifluoroacetic acid 0.05% in water to remove contaminants that could potentially disrupt the efficiency of the mass spectrometry analysis. After 6 min, the concentration column was put in line with a nano debit analytical column operating at 400 nL/min. The peptides were then separated according to their hydrophobicity (column C18, length 25 cm, diameter 75 μm, SN 10711310), using a gradient of a solution of acetonitrile (ACN/FA-99.9/0.1) of 4 to 25% in 50 min.

#### 2.4.3. LC-MS/MS Data Processing and Protein Identification

The raw files from the LC-MS/MS were aligned against the *Bos taurus* database (i.e., ref_bos_taurus, 23,970 sequences) with Mascot V.2.5.1 (http://www.matrixscience.com, accessed on 30 August 2020). The precursor and fragment mass tolerance were set up at 10 ppm and 0.02 Da, respectively. The variable modifications included carbamidomethylation (C), oxidation (M), and deamidation (NQ). Protein identification could be verified when at least two peptides derived from one protein showed statistically significant identity. The Mascot score was 33 with a False Discovery Rate of 1%, and the *p*-value was adjusted at a given threshold (0.0093).

### 2.5. Bioinformatics Analyses

#### 2.5.1. Protein-Protein Interactions (PPI)

The protein-protein interactions between the putative protein biomarkers were analysed using the STRING web service database (https://string-db.org/, accessed on 28 November 2020). Default settings were used, i.e., medium confidence of 0.4 and 4 criteria for linkage: co-occurrence, experimental evidence, existing databases, and text mining. As the bovine Gene Ontology (GO) had limits, orthologous human Uniprot IDs, following the procedure by Gagaoua et al. [14], were used for this analysis to take advantage of the most complete annotations available.

#### 2.5.2. Gene Ontology and Pathway and Process Enrichment Analyses

The pathway and Gene Ontology analyses were performed using two web-based tools. First, ProteINSIDE (http://www.proteinside.org/, accessed on 28 November 2020) was used to investigate GO terms for potential functions and molecular mechanisms [23]. For this analysis, the top 20 GO enrichment terms (*p*-value, Benjamini–Hochberg < 0.05) were considered and covered Biological Process (BP), Molecular Function (MF), and Cellular Component (CC) categories. The Metascape^®^ (https://metascape.org/, accessed on 28 November 2020) web service tool was further used to investigate the pathway and process enrichment analyses using the list of 34 differential proteins. The statistically significant enriched ontology terms were displayed based on the hypergeometric test and Benjamini–Hochberg *p*-value correction algorithm [24].

### 2.6. Statistical Analyses

Statistical analyses of protein abundance were performed with XLSTAT 2018.2 (AddinSoft, Paris, France), as well as the online tools NormalyzerDE and MetaOmGraph, mainly for data standardisation. Raw data were scrutinised for data entry errors, any missing data, or outliers. Log2 transformation and mean normalisation were performed on protein abundance among replicate samples. For the comparison of protein abundance between the tender (low WBSF values) and tough meat samples (high WBSF values), a one-way analysis of variance was performed for each protein. Differences in protein abundance between the tender and tough groups were considered significant at *p* < 0.05, and significant proteins were considered as candidate protein biomarkers. Pearson correlations were computed between the individual WBSF values and protein abundances for those proteins significant following ANOVA. Correlations were considered significant at *p* < 0.05. To get an overview of the main proteins related to WBSF variability, Partial Least Squares (PLS) regressions on standardised data were conducted to generate explanatory models using the list of the candidate protein biomarkers and identify the most influential proteins based on the variable importance in projection (VIP) filter set at both VIP > 1.0 and >0.8, as described by Gagaoua et al. [9]. Moreover, a stepwise regression analysis was used to explain WBSF using the 34 differential proteins (as independent variables, x). The absence of collinearity was systematically tested [25], specifically, the variable was identified as collinear if it possessed a high condition index > 10. The regression model allowed the entry of no more than 3 explanatory variables based on the parsimony principle.

## 3. Results

### 3.1. Differential Proteins between Extreme Groups of High and Low WBSF Values

According to Huffman et al. [26], for beef cooked to 70 °C, meat with Warner–Bratzler shear force values of 4.1 kg (40.18 N) or less was correlated with high levels (98%) of consumer acceptability, while beef prepared under the same conditions with shear force values of 5.8 kg (56.84 N) or greater remained unacceptable. Two groups of beef samples with a large difference in shear force were selected from a panel of 107 beef animals collected and profiled under similar conditions. The mean shear force value in the lower shear force group was 33.21 N, while the mean shear force value for the other group was 63.96 N. These groups were classified as tender and tough, respectively. Putative protein biomarkers of beef tenderness that significantly differed in abundance in muscle samples were identified from these divergent groups (Table 1 and details in Table S1).

A total of 34 proteins were different (*p* < 0.05) in their abundance between the tender and tough groups (Table 2). These 34 proteins belonged to five major biological pathways (Table 2), these being: (i) muscle contraction, structure, and associated proteins (n = 17; 50%); (ii) energy metabolism and associated pathways (n = 5; 15%); (iii) heat shock proteins (n = 4; 12%); (iv) oxidative stress (n = 2; 6%); and (v) other pathways including regulation of cellular processes, binding, apoptotic, and transport proteins (n = 6; 17%). The 34 proteins were then compared with a database of beef tenderness biomarkers by Gagaoua et al. [13], of which 23 overlapped with the database (Table 2).

**Figure 1:**
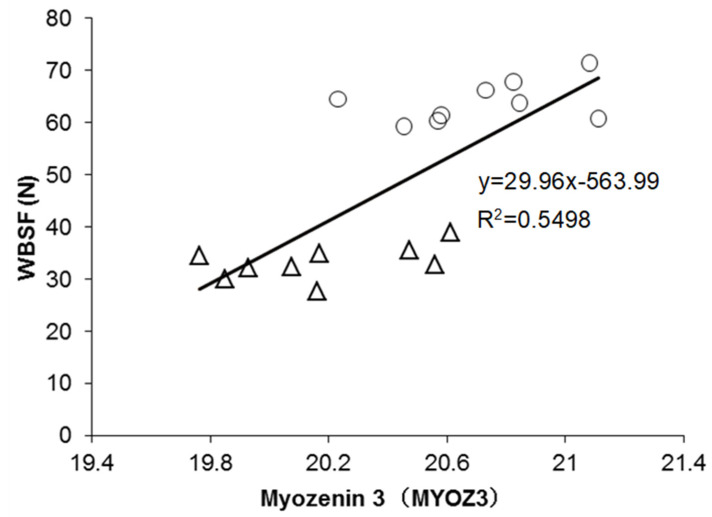
Example of significant correlations between the abundance of Myozenin 3 (MYOZ3) and WBSF values. The tender samples are shown by triangles (∆) and the tough samples by circles (○). The R-square of the correlation is given.

The regression model built for WBSF is presented in Table 3. The model explained 79% of the variability in WBSF (*p* < 0.01), including the abundance of three proteins: MYOZ3 (Myozenin 3), BIN1 (Bridging Integrator-1), and OGN (Mimecan), which were all positively correlated with WBSF (negatively with tenderness). It should be highlighted that MYOZ3 alone explained 52% of the variability. In this model, the correlation of MYOZ3 with WBSF values is depicted in Figure 1.

**Figure 2:**
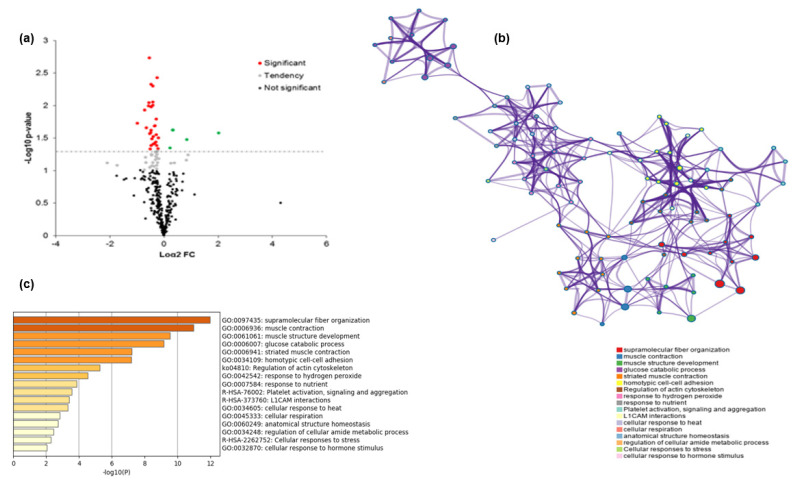
Bioinformatics and statistical analyses of the proteins identified to be differential between the tough and tender *Longissimus thoracis* muscle steaks. (**a**) Volcano plot of the differential proteins in terms of their abundance, with a total of 34 proteins that were significantly different between the two tenderness groups shown in red (negative direction with tenderness) and green colour (positive direction with tenderness). The other proteins that had a tendency or were not significant were in grey and black colour, respectively. (**b**) Networks of pathways and process enrichment cluster analysis based on the 34 differential proteins using Metascape® (https://metascape.org/, accessed on 28 November 2020). (**c**) Functional enrichment analysis based on the list of significant 17 Gene Ontology (GO) terms ranked by their *p*-value.

From the list of the putative protein biomarkers, 30 were negatively correlated with tenderness (positively with WBSF), from which MYOZ3, CFL2, and BIN1 were the most highly significantly correlated proteins. In addition, 4 proteins (COL1A1, ADSL, HSPD1, and PRDX3) were positively correlated with tenderness (negatively with WBSF; Figure 2a and Table 2). From the correlation analyses, Myozenin (MYOZ3) was strongly and significantly correlated with WBSF (Figure 1). No significant correlation was found between WBSF with KLHL41 and VCL (Table 2).

### 3.2. Partial Least Squares to Explain the Variability of WBSF Values

Based on the VIP filter (Table 4), the WBSF PLS regression model retained 32 proteins, of which 16 proteins (MYOZ3, FHOD1, CFL2, PDLIM7, TMOD4, LGALS1, TPI1, ADSL, ALDOC, TRIM72, BIN1, MYLPF, CORO6, HSPA8, OGN, EIF5A) had a VIP > 1.0. The other 16 proteins (HSPD1, MYL1, ALDOA, WDR1, PKM, COL1A1, HSPB1, APOBEC2, ACTN3, ACTB, PDLIM3, CAMK2D, PARK7, PRDX3, STIP1, RDX) had a VIP between 0.8 and 1.0; KLHL41 and VCL were the only two proteins whose VIP values were under 0.8. Combined with the results of the correlation analyses, the 32 proteins were identified as related to WBSF regardless of the statistical method. In addition, MYOZ3 was the first ranked protein, with the highest VIP.

### 3.3. Protein-Protein Interactions (PPI)

**Figure 3:**
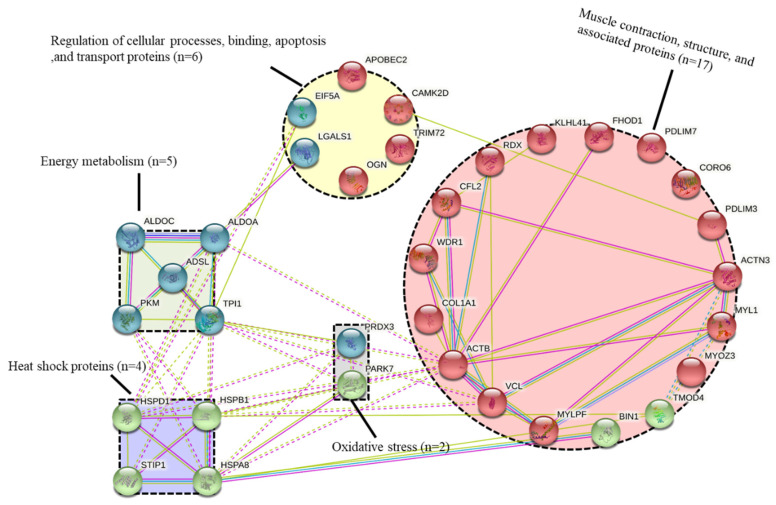
Protein-Protein interaction network built using the 34 differentially abundant proteins. The interaction map was generated from the web-based search STRING database (https://string-db.org/, accessed on 28 November 2020). Default settings of confidence of 0.6 and 4 criteria for linkage: co-occurrence, experimental evidence, existing databases, and text mining were used.

The protein-protein interaction network highlighted the importance of the structural and contractile pathways in beef tenderisation (Figure 3). In the network, ACTB (Actin) had the most interactions with other pathways, including energy metabolism and heat shock proteins, while ACTN3 (Alpha-actinin-3) and MYLPF (Myosin regulatory light chain 2) had more involvement within the muscle structure pathway. The next most dominant pathways were cellular processes, binding, apoptosis, and transport proteins, which showed multiple interactions with the energy metabolism (TPI1, ALDOA, and PKM), heat shock proteins (HSPD1), and muscle contraction (PDLIM3). It should be noted that the proteins in the heat shock pathway had a close interaction with each other.

### 3.4. Pathway and Process Enrichment Analysis

The Gene Ontology (GO) results are given in Table 5. Canonical glycolysis (GO:0061621), glycolytic process (GO:0006096), and muscle contraction (GO:0006936) were the top three Gene-Ontology (GO)-enriched terms identified from the list of the 34 differential proteins (Table 5), while Cellular Component (CC), cytosol (GO:0005829), extracellular exosome (GO:0070062), and cytoplasm (GO:0005737) were the most important three CC terms. It should be noted that a considerable number of proteins were classified as proteins binding (GO:0005515) in molecular function (Table 5). From the Metascape analysis, 17 top and significantly enriched terms were validated and allowed to construct process enrichment networks of the pathways (Figure 2b,c). The top six enriched term clusters were highlighted, including supramolecular fibre organisation (GO:0097435), muscle contraction (GO:0006936), muscle structure development (GO:0061061), glucose catabolic process (GO:0006007), striated muscle contraction (GO:0006941), and homotypic cell-cell adhesion (GO:0034109). The most dominant pathway was supramolecular fibre organisation and muscle contraction, which was consistent with the PPI data confirming their pivotal role in beef tenderisation of young Limousin bull beef (Figure 3).

## 4. Discussion

The beef industry is consistently confronted with challenges in supplying beef with consistent eating qualities. Tenderness is one of the most important palatability traits of beef that affects the repurchase decisions of consumers. The pathways underpinning beef tenderness determination are complex and not fully elucidated, although a recent integromics meta-analysis by Gagaoua et al. [13] on the molecular signatures shed light on some of them. Thus, it was valuable to identify putative protein biomarkers of beef tenderness from two tenderness groups with a strong difference in shear force: tender (33.21 N) vs. tough (63.96 N; Table 1).

This study on Irish Limousin-cross cattle allowed us to get more insights and validate the association of certain proteins with tenderness and propose new ones that will further increase our knowledge and progress in the pipeline of beef tenderness discovery of biomarkers. This study allowed us also to (i) propose preliminary explanatory models of tenderness using multiple regression and partial least squares; (ii) compare the list of putative protein biomarkers identified in this trial with previous studies to verify the robustness of the discovered proteins; and finally, (iii) increase our knowledge on the biological pathways involved in the variation of beef tenderness evaluated in this study using WBSF at an end-point cooking temperature of 71 °C. The relationship between tenderness and the list of candidate proteins was discussed in the following sections.

### 4.1. The Best Explanatory Proteins in the Regression Model of WBSF

The best regression model built with MYOZ3, BIN1, and OGN proteins explained 79% of the observed variability in WBSF (*p* < 0.01). MYOZ3 is mainly expressed in skeletal muscle and enriched in fast-twitch muscle fibres. MYOZ3 belongs to the myozenin family, of which three other members were previously proposed as tenderness biomarkers [13]. MYOZ3 acts as an intracellular binding protein to link with Z-disc proteins such as alpha-actinin and gamma-filamin and transmit calcineurin signalling to the sarcomere [27]. Due to the capacity to bind multiple proteins, the relationship between MYOZ3 and meat quality, specifically tenderness, could be through regulating the Z-disc structure and signal transduction, influencing muscle fibre differentiation [28]. Consistent with our findings, a previous study reported a negative association between MYOZ3 and the shear force of *M. longissimus thoracis* in heifers [29].

BIN1, also known as Bridging Integrator-1, was identified in the present study for the first time to have a potential association with beef tenderness. BIN1 plays an important role in the regulation of endocytosis and has other roles as a central regulator of cell proliferation and apoptosis [30]. While no evidence was present in the literature on a specific relationship with tenderness, BIN1 was associated with another important beef production attribute, residual feed intake [31], which was previously associated with meat quality. OGN, which is also called mimecan, belongs to a secreted protein family of small leucine-rich proteoglycans located in the extracellular matrix [32]. OGN was negatively correlated with beef tenderness, and both MYOZ3 and OGN genes were located within a Quantitative Trait Loci (QTL) for shear force on chromosome 8 (Table 2). Interestingly, when protein profiles were compared between Japanese Black cattle and Holstein cattle, a higher abundance of OGN protein (mimecan) was found in the Holstein breed known to have lower fat content [33]. An important function of OGN is in collagen fibrillogenesis [32]. For this reason, it could be hypothesised that the greater abundance of OGN protein observed for tougher beef animals may be related to a higher abundance of connective tissue content in the muscle of tough beef [34], although we did not measure the connective tissue content in the present study.

### 4.2. Dominant Pathway Related to WBSF of Young Limousin-Sired Bulls

Muscle contraction and structure were identified as the most important pathway associated with WBSF in this study. Most of the proteins from this pathway were localised in the sarcomere. Of these, compared with the database of beef tenderness biomarkers of Gagaoua [13], 13 were already identified, and 4 proteins (BIN1, FHOD1, CORO6, and RDX) were reported for the first time in this study.

Myosin and actin were critically important to textural changes in muscle that occurred post-mortem during meat ageing through the weakening of the actin/myosin complex in the myofibril [22]. As the major component of the thick filaments of the myofibril, molecular myosin consisted of two heavy and four light chains. This study revealed, for example, MYLPF (Myosin regulatory light chain 2) and MYL1 (Myosin light chain 1/3) to be negative biomarkers of beef tenderness. Myosin light chains were wrapped around the head/rod junction of the myosin heavy chain in skeletal muscle myosin [35]. The MYLPF and MYL1 proteins were demonstrated [13] to correlate with beef tenderness; however, the direction of their relationships with this trait lacks consistency across studies; this phenomenon was well known to vary depending on the breed and muscle type [5,25,36], and was suggested to be related to post-translational modifications of the proteins [37]. Myosin light chain proteins were highly expressed in fast-twitch fibres. It was noteworthy that phosphorylation of MYL might play an essential role in proteolysis and onset of apoptosis in post-mortem muscle, which was favourable to the degradation of large molecules and final tenderisation of aged meat [38]. As for the second, most abundant myofibrillar protein in muscle, actin was interrelated with the apoptosis of the cytoskeleton during meat tenderisation [39]. In our study, ACTB, ACTN3, and TMOD4 were negatively correlated with beef tenderness, which is consistent with previous studies [9,29].

The collagen alpha-1(I) chain is an abundant connective tissue protein with an important function of support in the muscle tissue and bone in the body, and is encoded by *COL1A1* gene. Several studies showed a close relationship between collagen content and variation in meat tenderness [40,41]. Interestingly, in a previous study by Bjarnadóttir et al. [42], COL1A1 and COL1A2 were found to have lower abundance in tender beef muscle, which was opposite to our findings. However, there was also evidence of a positive relationship between COL1A1 abundance and intramuscular fat content, which could have an effect in promoting beef tenderness [43]. Thus, there was no consistent conclusion regarding the direct influence of COL1A1 on meat quality.

PDLIM3 and PDLIM7 are two members of the PDZ and LIM domain (PDLIM) family, participating in multifunctional protein-protein interaction, cytoskeleton, and signal transduction pathways [44]. PDLIM family proteins contain a PDZ domain in the N-terminal portion and the LIM domain in the C-terminal portion [45]. PDLIM1 and PDLIM7 were previously identified as negative biomarkers of beef tenderness [13], which was consistent with our results stating that PDLIM3 and PDLIM7 were positively correlated with WBSF and the negative relationship of this protein family with beef quality.

Of the putative biomarkers identified for the first time in this study, FHOD1 is an actin regulator which played an important role in the stabilisation of filamentous (F)-actin bundles by selectively covering and binding their barbed ends to actin filaments, thus protecting actin filaments in cytoskeletal structures [46]. Likewise, CORO6 is also an actin-binding protein that is mainly expressed in the heart and skeletal muscle [47]. RDX (Radixin) is referred to as a member of ERM (Ezrin/Radixin/Moesin) proteins which help maintain cytoskeletal organisation by binding specific membrane proteins to the actin cytoskeleton [48]. FHOD1, CORO6, and RDX were all positively correlated with WBSF (and negatively with tenderness), which could be related to their protective effect on the integrity of the cytoskeleton.

### 4.3. Candidate Protein Biomarkers of WBSF from the Energy Metabolism Pathway

Energy metabolism comprises a series of interconnected pathways that can function in the presence or absence of oxygen to generate adenosine triphosphate (ATP), which is an end-product of the processes of oxidative phosphorylation [49]. Of the five proteins identified from this pathway (TPI1, ALDOA, PKM, ADSL, and ALDOC), the first four proteins have been previously identified as putative biomarkers of beef tenderness. In this study, these proteins were all negatively correlated with beef tenderness except ADSL. TPI1 can catalyse the conversion of dihydroxyacetone phosphate to D-glyceraldehyde 3-phosphate, meanwhile, maintaining the equilibration of the triosephosphates produced by aldolase (ALDOA) [50]. Aldolase is an enzyme that catalyses the reversible conversion of fructose-1,6-bisphosphate to glyceraldehyde 3-phosphate and dihydroxyacetone phosphate [51]. ALDOA and ALDOC are two different isoformes of aldolase. In the literature, ALDOA was both positively and negatively correlated with beef tenderness depending on the gender and muscle fibre type [13], while ALDOC was first identified as a putative negative biomarker of tenderness in the present study. The relationship between aldolase and tenderness could be explained by its participation in muscle glycolysis, which, if variable, could alter the profile and extent of pH decline, thereby further influencing the integrity of the Z-line with consequences for beef tenderness [52].

Pyruvate kinase, also known as PKM, is an enzyme that catalyses the dephosphorylation of phosphoenolpyruvate to pyruvate, generating ATP and regulating cell metabolism during glycolysis [53]. PKM1 and PKM2 are the two predominant isoforms of PKM in skeletal muscles. PKM was listed in the repertoire of beef tenderness biomarkers in *longissimus* muscle [13], and our findings provided further corroboration for its role in beef tenderness determination.

### 4.4. Heat Shock Proteins (HSPs) as Important Indicators of WBSF

Heat shock proteins (HSPs) are a family of proteins that have as their main function the protection of the organism itself and its cellular structures in response to exposure to stressful conditions [54]. The current study showed a differential abundance of HSPA8, HSPD1, HSPB1, and STIP1, three of which were already discovered to play a role in the variability of tenderness. Interestingly, three of the HSPs identified here were from three different subfamilies of HSPs, i.e., the small HSPs (HSPB1), HSP70s (HSPA8), and HSP60s (HSPD1). Among those four proteins, HSPA8, HSPB1, and STIP1 showed higher abundance in tough meat while HSPD1 was in the opposite direction.

As one important member of the large HSP70 family, HSPA8 was identified in six previous studies to be related to beef tenderness, but the mechanistic connection with tenderness was not clear because the protein was sometimes positively correlated and sometimes negatively correlated with beef tenderness [13]. The impact of HSP70 proteins on meat tenderness was thought to be mainly because they obstruct pro-apoptotic factors such as Bcl-2 in apoptotic pathways [13,55]. HSPA8 is grouped in the response to unfolded protein (GO:0006986) and protein refolding (GO:0042026) in Table 5. In this sense, HSPA8 played an important role in response to cellular stress [56]. Moreover, this protective role might be based on its interaction with structural proteins or by regulating cell signalling pathways (Figure 3). STIP1, known as a stress-induced-phosphoprotein, is a co-chaperone whose negative relationship with tenderness, found here, was consistent with the findings of Picard and Gagaoua [8].

As for the small HSPs, HSPB1 was identified as a robust biomarker of beef tenderness (referring to the database by Gagaoua et al. [13]), and it was, from that integromics study, in the top five biomarkers of beef tenderness from a list of 124 proteins. Extrinsic stressors, such as pre-slaughter or post-mortem management conditions, were sources of the intensive production of sHSPs in the muscle, which, like the larger HSPs, also play a regulatory role in delaying the apoptosis onset, the protection of myofibrillar proteins from proteolysis, and other cellular homeostasis roles [15,39]. The positive and negative relationships identified between sHSPs and tenderness might be due to interactions of factors such as animal type/breed, gender, muscle type, and pre-slaughter conditions [12,39,57]. In this study, HSPB1 was negatively correlated with beef tenderness, which would be consistent with its protective function against proteolysis in skeletal muscle.

It was notable that HSPD1, which is a member of the HSP60 family, was identified to be correlated with beef tenderness for the first time in the present study. Under stress conditions, the HSP60 family of proteins inside the mitochondrial matrix usually acts as molecular chaperones, collaborating with the co-chaperone Hsp10 to promote the correct folding of imported proteins and proper assembly of unfolded polypeptides [58]. A positive relationship between HSPD1 and tenderness might be hypothesised by its function in the energy metabolism pathway to maintain energy supply during proteolysis of myofibril proteins (Figure 3). As with HSPB1, HSPD1 was also associated with beef colour, which deepened our knowledge of the influential role of HSP proteins in post-mortem muscle events and consequences on meat quality [14].

### 4.5. Putative Biomarkers of Tenderness Related to Oxidative Stress

After slaughter, the lipid and protein fractions of muscle are targeted by various reactive oxygen species (ROS), causing structural alteration or denaturation of proteins [59]. In the context of oxidative stress, meat tenderness can be affected by cellular antioxidants, which include both enzymatic and non-enzymatic scavenger agents engaged in protecting the muscle proteins from damage by ROS, thereby further maintaining cell homeostasis. Meanwhile, it is noteworthy that meat tenderness could also be influenced by ROS damage produced by mitochondria, which play an important role in supplying energy during the conversion of muscle into meat [60,61].

In this study, two important proteins from the oxidative pathway, i.e., PARK7 and PRDX3 were identified and validated in comparison to the previous list of robust beef tenderness biomarkers [13]. PARK7, also named DJ-1, was secreted from the cytosol to mitochondria to remove the mitochondrial H_2_O_2_ and maintain the integrity of the organelle in response to oxidative stress [62]. Consistent with the results of most previous studies, PARK7 level was negatively correlated with beef tenderness [8,63,64]. A mechanism could be deduced where PARK7 had an inhibitory effect on the pro-apoptotic factors and caspases (proteolytic enzymes) by interacting with other proteins from energy metabolism and HSPs pathways, as depicted by the PPI network (Figure 3), thus contributing to beef toughness by slowing or limiting post-mortem apoptosis of muscle cells [13,65].

PRDX3 is a member of the peroxiredoxins (Prxs), a ubiquitous family with six subgroups, and which, in bovine, contains six members [66]. PRDX3 is exclusively located in mitochondria with an oligomeric ring structure [67]. It should be highlighted that PRDX3 was previously identified to be positively related to beef tenderness in *Semimembranosus* muscle [68], which was consistent with our findings for *Longissimus thoracis* muscle. Regarding the possible mechanism, it could be assumed that the antioxidant enzyme PRDX3 could prevent the accumulation of ROS, protecting the function of proteases and the operation of the electron transport system, and thus, leading to apoptosis promotion and meat tenderisation. Other members of peroxiredoxins were also found to be associated with several beef quality traits, including PRDX1 [69] and PRDX2 [70] with tenderness and PRDX6 with tenderness [9], pH decline [71], and beef colour [25].

### 4.6. Proteins from Other Pathways

LGALS1 and TRIM72 were negative biomarkers of beef tenderness in this study, which was consistent with previous reports [8,9,42]. LGALS1 (Galectin-1) belongs to a family of β-galactoside-binding proteins, which may act as promoters of apoptosis and have an impact on cell proliferation and skeletal muscle differentiation [42]. However, the mechanism behind the association between Galectin-1 and meat tenderness was still obscure due to its complex functions under different conditions. As a signalling protein expressed in skeletal muscle, Tripartite motif-containing 72 (TRIM72) was considered as a sensor of oxidation on membrane damage [72]. TRIM72 may act as a scavenger of the harmful agents accumulated under the apoptotic process, leading to a limitation of apoptosis and tough meat [9]. In line with our findings, there was also a higher abundance of TRIM72 reported in tough beef, hence showing its negative role in the apoptotic pathway [68]. In addition, TRIM72 was first identified to correlate with beef colour, which confirmed the anti-oxidative properties of this protein, allowing it to be suggested as a relevant marker for multiple beef quality traits [63].

## 5. Conclusions

The results of this study allowed us to validate 23 putative biomarkers on Irish cattle (Limousin-sired bulls) and to propose 11 new proteins that increased our knowledge on the main biological pathways underpinning beef tenderness variation in the *Longissimus thoracis* muscle of young bulls. The network and gene ontology analyses allowed us to better characterise the enriched molecular pathways. This study also suggested a regression model with an R-squared of 79% using three proteins-MYOZ3, BIN1, and OGN-to explain the relationship between the abundance of these protein biomarkers and WBSF values. Further analyses would assess the robustness of the list of putative biomarkers identified in this study using accurate methods and new populations.

## Figures and Tables

**Table 1:** Warner–Bratzler shear force (WBSF) values of the *Longissimus thoracis* muscles used in this trial.

Quality Traits	Min	Max	Mean	SD	CV (%)
WBSF (N) (n = 9)	27.70	38.85	33.21	3.24	9.75
WBSF (N) (n = 9)	59.25	71.40	63.96	3.98	6.22

N, Newtons; SD, Standard Deviation; CV, Coefficient of Variation.

**Table 2:** List of the 34 differential proteins organised by biological family, identified to significantly differ among the two WBSF (tenderness) groups.

Uniprot ID	Gene Name	Full Protein Name	Differences		Pearson Correlations ^a^	Overlap with Gagaoua et al. Database [13]
			Fold Change (Log2)	*p*-Value	WBSF	
**Muscle contraction, structure and associated proteins (n = 17)**						
Q08DI7	MYOZ3 ^b^	Myozenin 3	−0.53	0.002	0.741 ***	✓
E1BNG8	BIN1	Bridging Integrator-1	−0.25	0.003	0.616 **	
Q148F1	CFL2	Cofilin-2	−0.47	0.003	0.670 **	
E1BIN0	FHOD1	Formin homology 2 domain containing 1	−0.40	0.005	0.714 ***	
A6QLZ8	CORO6	Coronin	−0.57	0.007	0.607 **	
Q0P571	MYLPF	Myosin regulatory light chain 2	−0.71	0.009	0.613 **	✓
Q3SX40	PDLIM7 ^b^	PDZ and LIM domain protein 7	−0.47	0.010	0.642 **	✓
Q0VC48	TMOD4	Tropomodulin-4	−0.42	0.010	0.640 **	TMOD1
Q2KJH4	WDR1	WD repeat-containing protein 1	−0.49	0.019	0.542 *	✓
P60712	ACTB	Actin, cytoplasmic 1	−0.98	0.023	0.524 *	✓
A0JNJ5	MYL1	Myosin light chain 1/3, skeletal muscle isoform	−0.47	0.024	0.554 *	✓
P02453	COL1A1	Collagen alpha-1(I) chain	0.89	0.025	−0.536 *	✓
Q3SYZ8	PDLIM3	PDZ and LIM domain protein 3	−0.51	0.029	0.512 *	PDLIM7/PDLIM1
Q0III9	ACTN3 ^c^	Alpha-actinin-3	−0.34	0.034	0.529 *	✓
A4FV78	KLHL41	KBTBD10 protein	−0.38	0.037		✓
F1N789	VCL	Vinculin	−0.21	0.040		✓
Q32LP2	RDX	Radixin	−0.26	0.043	0.476 *	
**Energy metabolism (n = 5)**						
Q5E956	TPI1	Triosephosphate isomerase	−0.35	0.018	0.631 **	✓
Q3ZBY4	ALDOC	Fructose-bisphosphate aldolase	−0.33	0.018	0.621 **	✓
A5D984	PKM	Pyruvate kinase	−0.39	0.029	0.540 *	✓
A6QLL8	ALDOA	Fructose-bisphosphate aldolase	−0.29	0.029	0.554 *	✓
A3KN12	ADSL	Adenylosuccinate lyase	0.27	0.019	−0.629 **	
**Heat shock proteins (n = 4)**						
P19120	HSPA8 ^b^	Heat shock cognate 71 kDa protein	−0.41	0.006	0.590 **	✓
P31081	HSPD1	60 kDa heat shock protein, mitochondrial	0.84	0.016	−0.567 *	
Q3T149	HSPB1	Heat shock protein beta-1	−0.44	0.038	0.531 *	✓
Q3ZBZ8	STIP1 ^b,c^	Stress-induced-phosphoprotein 1	−0.29	0.040	0.484 *	✓
**Oxidative stress (n = 2)**						
P35705	PRDX3	Thioredoxin-dependent peroxide reductase	0.32	0.038	−0.488 *	PRDX6/PRDX1/PRDX2
Q5E946	PARK7	Protein/nucleic acid deglycase DJ-1	−0.47	0.046	0.501 *	✓
**Other pathways (n = 6)**						
P11116	LGALS1	Galectin-1	−0.56	0.006	0.639 **	✓
E1BE77	TRIM72	Tripartite motif containing 72	−0.45	0.008	0.620 **	✓
P19879	OGN ^b^	Mimecan	−0.64	0.017	0.589 *	
Q2HJF7	CAMK2D	Calcium/calmodulin-dependent protein kinase	−0.20	0.038	0.510 *	
Q6EWQ7	EIF5A	Eukaryotic translation initiation factor 5A-1	−0.45	0.046	0.574 *	
Q3SYR3	APOBEC2	Probable C->U-editing enzyme APOBEC-2	−0.66	0.047	0.530 *	

^a^ Significance of the correlations: * *p* < 0.05; ** *p* < 0.01; *** *p* < 0.001. ^b^ Proteins identified as Quantitative Trait Loci (QTL) of shear force using ProteQTL tool included in ProteINSIDE (http://www.proteinside.org/, accessed on 28 November 2020) from the Animal QTL Database (https://www.animalgenome.org/QTLdb/, accessed on 28 November 2020). ^c^ Proteins identified as QTL of sensory tenderness.

**Table 3:** Best regression equation of WBSF based on the list of the significant differential proteins from Table 2.

R-Squared ^a^	S.E	Entered Independent Variable ^b^	Partial R-Squared	Regression Coefficient	t-Value	*p*-Value
0.79 **	0.125	MYOZ3	0.52	0.486	3.875	0.002
	0.116	BIN1	0.17	0.454	3.907	0.002
	0.121	OGN	0.1	0.347	2.868	0.012

^a^ Significance of the models: ** *p* < 0.01. ^b^ Variables are shown in order of their entrance, in a stepwise manner, in the regression model.

**Table 4:** Partial Least Squares (PLS) prediction of beef tenderness (WBSF) using the list of the 34 putative protein biomarkers based on their variable importance in the projection (VIP).

Proteins	VIP	Direction (+ or −)
MYOZ3: Myozenin 3	1.291	−
FHOD1: Formin homology 2 domain containing 1	1.243	−
CFL2: Cofilin-2	1.168	−
PDLIM7: PDZ and LIM domain protein 7	1.119	−
TMOD4: Tropomodulin-4	1.115	−
LGALS1: Galectin-1	1.113	−
TPI1: Triosephosphate isomerase	1.099	−
ADSL: Adenylosuccinate lyase	1.095	+
ALDOC: Fructose-bisphosphate aldolase	1.082	−
TRIM72: Tripartite motif containing 72	1.080	−
BIN1: Bridging Integrator-1	1.073	−
MYLPF: Myosin regulatory light chain 2, skeletal muscle isoform	1.067	−
CORO6: Coronin	1.056	−
HSPA8: Heat shock cognate 71 kDa protein	1.027	−
OGN: Mimecan	1.025	−
EIF5A: Eukaryotic translation initiation factor 5A-1	1.000	−
HSPD1: 60 kDa heat shock protein, mitochondrial	0.987	+
MYL1: Myosin light chain 1/3, skeletal muscle isoform	0.965	−
ALDOA: Fructose-bisphosphate aldolase	0.965	−
WDR1: WD repeat-containing protein 1	0.943	−
PKM: Pyruvate kinase	0.940	−
COL1A1: Collagen alpha-1(I) chain	0.934	+
HSPB1: Heat shock protein beta-1	0.924	−
APOBEC2: Probable C->U-editing enzyme APOBEC-2	0.923	−
ACTN3: Alpha-actinin-3	0.922	−
ACTB: Actin, cytoplasmic 1	0.913	−
PDLIM3: PDZ and LIM domain protein 3	0.892	−
CAMK2D: Calcium/calmodulin-dependent protein kinase type II subunit delta	0.889	−
PARK7: Protein/nucleic acid deglycase DJ-1	0.873	−
PRDX3: Thioredoxin-dependent peroxide reductase, mitochondrial	0.850	+
STIP1: Stress-induced-phosphoprotein 1	0.843	−
RDX: Radixin	0.830	−
KLHL41: KBTBD10 protein	0.771	−
VCL: Vinculin	0.708	−

**Table 5:** Top20 Gene Ontology (GO) terms computed using the list of the 34 putative protein biomarkers.

GO	Function	Gene Name	GO Frequency within the Dataset (%)	GO Frequency within the Genome (%)	*p*-Values
**Biological Process (BP)**					
GO:0061621	canonical glycolysis	ALDOC TPI1 PKM ALDOA	11.76	14.81	2.13 × 10^−9^
GO:0006096	glycolytic process	ALDOC ALDOA PKM TPI1	11.76	10.26	5.4 × 10^−9^
GO:0006936	muscle contraction	TRIM72 MYL1 VCL MYLPF TMOD4	14.71	2.35	2.86 × 10^−8^
GO:0043312	neutrophil degranulation	VCL HSPA8 ALDOA ALDOC PKM	14.71	1.03	1.28 × 10^−6^
GO:0070527	platelet aggregation	ACTB VCL HSPB1	8.82	7.14	2.06 × 10^−6^
GO:0006094	gluconeogenesis	ALDOC TPI1 ALDOA	8.82	6.82	2.27 × 10^−6^
GO:0006986	response to unfolded protein	HSPB1 HSPA8 HSPD1	8.82	6.25	2.62 × 10^−6^
GO:0035633	maintenance of blood-brain barrier	VCL ACTB	5.88	66.67	5.99 × 10^−6^
GO:0030388	fructose 1,6-bisphosphate metabolic process	ALDOA ALDOC	5.88	28.57	1.98 × 10^−5^
GO:0030042	actin filament depolymerisation	WDR1 CFL2	5.88	25	2.34 × 10^−5^
GO:0043297	apical junction assembly	VCL WDR1	5.88	25	2.34 × 10^−5^
GO:0002576	platelet degranulation	ALDOA WDR1 VCL	8.82	2.44	3.11 × 10^−5^
GO:0030836	positive regulation of actin filament depolymerisation	CFL2 WDR1	5.88	15.38	4.82 × 10^−5^
GO:0006000	fructose metabolic process	ALDOA ALDOC	5.88	13.33	5.97 × 10^−5^
GO:0007015	actin filament organisation	ALDOA CORO6 TMOD4	8.82	1.54	9.6 × 10^−5^
GO:0042026	protein refolding	HSPD1 HSPA8	5.88	9.52	9.98 × 10^−5^
GO:0030239	myofibril assembly	KLHL41 TMOD4	5.88	7.14	0.000162
GO:0034333	adherens junction assembly	ACTB VCL	5.88	5.88	0.000221
GO:0043066	negative regulation of apoptotic process	HSPD1 HSPB1 PRDX3 PARK7	11.76	0.49	0.000221
GO:0086091	regulation of heart rate by cardiac conduction	BIN1 CAMK2D	5.88	5.56	0.000239
**Cellular Component (CC)**					
GO:0005829	cytosol	VCL TPI1 MYLPF HSPA8 STIP1 EIF5A CAMK2D ADSL MYL1 ACTN3 ALDOC HSPD1 BIN1 PRDX3 PARK7 PDLIM3 ALDOA HSPB1 WDR1 PKM FHOD1 PDLIM7 ACTB KLHL41	70.59	0.5	3.03 × 10^−21^
GO:0070062	extracellular exosome	ALDOC HSPD1 ACTN3 ACTB PARK7 TPI1 PKM VCL RDX CFL2 LGALS1 OGN ALDOA HSPA8 HSPB1 WDR1	47.06	0.58	1.67 × 10^−15^
GO:0005737	cytoplasm	PARK7 FHOD1 COL1A1 EIF5A PKM BIN1 HSPD1 HSPA8 KLHL41 PRDX3 TRIM72 APOBEC2 CAMK2D ACTB HSPB1 CFL2 LGALS1	50	0.41	3 × 10^−14^
GO:0005615	extracellular space	HSPD1 RDX ALDOA CFL2 HSPA8 TPI1 OGN COL1A1 HSPB1 LGALS1 ACTB	32.35	0.77	3 × 10^−12^
GO:0005925	focal adhesion	VCL HSPB1 RDX HSPA8 ACTN3 ACTB PDLIM7	20.59	1.82	2.33 × 10^−10^
GO:0015629	actin cytoskeleton	CFL2 MYOZ3 BIN1 PDLIM7 ACTB ALDOA	17.65	3.14	2.54 × 10^−10^
GO:0005856	cytoskeleton	VCL KLHL41 HSPB1 BIN1 TMOD4 ACTB FHOD1 ALDOC	23.53	1.08	3.05 × 10^−10^
GO:0030018	Z disc	PDLIM7 MYOZ3 BIN1 CFL2 PDLIM3	14.71	4.2	2.57 × 10^−9^
GO:1904813	ficolin-1-rich granule lumen	ALDOC HSPA8 PKM ALDOA VCL	14.71	4.03	2.92 × 10^−9^
GO:0005634	nucleus	STIP1 CAMK2D EIF5A BIN1 FHOD1 ALDOA PKM HSPB1 APOBEC2 ACTB HSPA8 PARK7 TPI1	38.24	0.26	4.61 × 10^−9^
GO:0005576	extracellular region	PKM ALDOC WDR1 LGALS1 ALDOA HSPA8 VCL COL1A1 OGN	26.47	0.49	7.91 × 10^−9^
GO:0034774	secretory granule lumen	ALDOC PKM VCL ALDOA HSPA8	14.71	1.56	2 × 10^−7^
GO:0031674	I band	ALDOA CFL2 BIN1	8.82	13.64	3.96 × 10^−7^
GO:0005912	adherens junction	PARK7 ACTB PDLIM3 PDLIM7 VCL	14.71	1.04	1.28 × 10^−6^
GO:0030864	cortical actin cytoskeleton	RDX CFL2 WDR1	8.82	8.57	1.28 × 10^−6^
GO:0001725	stress fibre	PDLIM3 PDLIM7 FHOD1	8.82	6.25	2.62 × 10^−6^
GO:0030424	axon	PARK7 HSPA8 BIN1 ACTB	11.76	1.65	3.52 × 10^−6^
GO:0005654	nucleoplasm	PARK7 CAMK2D ACTB KLHL41 HSPA8 FHOD1 PDLIM7	20.59	0.24	3.81 × 10^−5^
GO:0101031	chaperone complex	STIP1 HSPA8	5.88	15.38	4.82 × 10^−5^
GO:0005886	plasma membrane	VCL HSPA8 BIN1 HSPD1 ACTB RDX WDR1 KLHL41	23.53	0.18	5.47 × 10^−5^
**Molecular Function (MF)**					
GO:0005515	protein binding	PRDX3 PARK7 ALDOA OGN MYOZ3 ACTN3 FHOD1 CAMK2D PKM HSPD1 TRIM72 COL1A1 CFL2 TMOD4 STIP1 ALDOC CORO6 BIN1 LGALS1 VCL EIF5A HSPA8 HSPB1 PDLIM3 TPI1 KLHL41 RDX ACTB PDLIM7	85.29	0.45	9.5 × 10^−24^
GO:0042802	identical protein binding	ALDOA PRDX3 TRIM72 PARK7 ACTB CAMK2D ADSL BIN1 HSPB1 APOBEC2 ACTN3 FHOD1 COL1A1	38.24	0.92	2.98 × 10^−15^
GO:0003723	RNA binding	HSPB1 HSPD1 LGALS1 EIF5A APOBEC2 HSPA8 PKM ALDOA STIP1 RDX	29.41	0.62	2.33 × 10^−10^
GO:0045296	cadherin binding	PARK7 HSPA8 VCL RDX ALDOA PKM	17.65	2.03	2.44 × 10^−9^
GO:0051015	actin filament binding	BIN1 FHOD1 WDR1 CFL2 CORO6	14.71	3.57	4.61 × 10^−9^
GO:0003779	actin binding	PDLIM3 VCL ALDOA PDLIM7 MYOZ3 RDX	17.65	1.48	1.01 × 10^−8^
GO:0008307	structural constituent of muscle	ACTN3 MYL1 MYLPF	8.82	6.52	2.48 × 10^−6^
GO:0004332	fructose-bisphosphate aldolase activity	ALDOA ALDOC	5.88	66.67	5.99 × 10^−6^
GO:0031625	ubiquitin protein ligase binding	HSPA8 HSPD1 VCL TPI1	11.76	1.36	6.77 × 10^−6^
GO:0051087	chaperone binding	BIN1 HSPD1 HSPA8	8.82	3.3	1.44 × 10^−5^
GO:0048156	tau protein binding	BIN1 ACTB	5.88	20	3.26 × 10^−5^
GO:0023026	MHC class II protein complex binding	PKM HSPA8	5.88	11.76	7.34 × 10^−5^
GO:0051371	muscle alpha-actinin binding	PDLIM3 PDLIM7	5.88	11.11	7.98 × 10^−5^
GO:0044183	protein folding chaperone	HSPA8 HSPB1	5.88	7.14	0.000162
GO:0042803	protein homodimerisation activity	PARK7 CAMK2D TPI1 HSPB1	11.76	0.53	0.000173
GO:0003697	single-stranded DNA binding	HSPD1 PARK7	5.88	2.06	0.001335
GO:0019901	protein kinase binding	HSPB1 ACTB PRDX3	8.82	0.54	0.001393
GO:0044325	ion channel binding	ACTN3 CAMK2D	5.88	1.82	0.001617
GO:0002020	protease binding	COL1A1 BIN1	5.88	1.61	0.001708
GO:0070626	(S)-2-(5-amino-1-(5-phospho-D-ribosyl)imidazole-4-carboxamido)succinate AMP-lyase (fumarate-forming) activity	ADSL	2.94	100	0.001708

## Data Availability

Data sharing is not applicable to this article.

## Supplementary Materials

The following are available online at https://www.mdpi.com/article/10.3390/foods10050952/s1, Table S1. Individual Warner-Bratzler shear force (WBSF) values of the *Longissimus thoracis* muscles from the 18 animals used in this trial.
